# Ovarian cancer proliferation and apoptosis are regulated by human transfer RNA methyltransferase 9-like via LIN9

**DOI:** 10.3892/ol.2021.12459

**Published:** 2021-01-12

**Authors:** Huai Mei Chen, Jia Wang, Ying Feng Zhang, Yan Hong Gao

Oncol Lett 14: 4461-4466, 2017; DOI: 10.3892/ol.2017.6750

Following the publication of the above paper, an interested reader drew to the authors’ attention that, in Fig. 1 on p. 4464, the left-most hTRM9L and LIN9 panels (representing the data from ovarian cancer tissues at ×200 magnification) appeared to show an area of overlap. The authors were able to consult their original data, and realized that the Figure had been compiled incorrectly; essentially, the wrong image was chosen for the LIN9 panel.

The revised version of Fig. 1, showing the correct data for the abovementioned panel, is shown below. This error did not have a significant impact on the results or the conclusions reported in this study. The authors are grateful to the Editor of *Oncology Letters* for allowing them the opportunity to publish this Corrigendum, and all of the authors agree to the publication of this Corrigendum. The authors sincerely apologize for this mistake, and regret any inconvenience this mistake has caused.

## Figures and Tables

**Figure 1.**
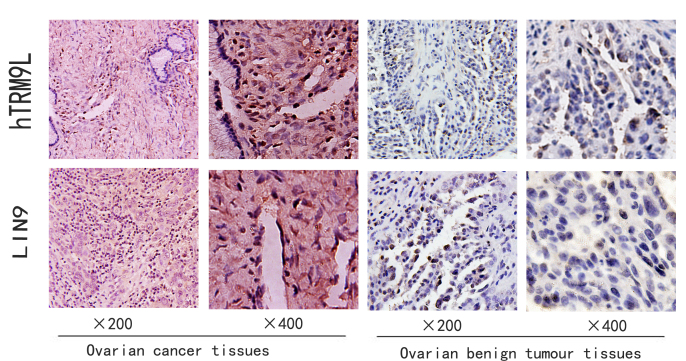
Immunohistochemical staining of hTRM9L and LIN9 in ovarian cancer and normal tissues. hTRM9L, human transfer RNA methyltransferase 9-like.

